# Colorectal Cancer Outcomes: A Comparative Review of Resource-Limited Settings in Low- and Middle-Income Countries and Rural America

**DOI:** 10.3390/cancers16193302

**Published:** 2024-09-27

**Authors:** Clare E. Jacobson, Calista M. Harbaugh, Kwabena Agbedinu, Gifty Kwakye

**Affiliations:** 1 Department of Surgery, University of Michigan, Ann Arbor, MI 48109, USA; 2 Center for Healthcare Outcomes and Policy, University of Michigan, Ann Arbor, MI 48109, USA; 3 Directorate of Surgery, Komfo Anokye Teaching Hospital, Kumasi 23321, Ghana; 4 Center for Global Surgery, University of Michigan, Ann Arbor, MI 48109, USA

**Keywords:** colorectal cancer, colon cancer, global surgery, LMIC, rural surgery, rural health, health disparities

## Abstract

**Simple Summary:**

Colorectal cancer is becoming more common in low- and middle-income countries (LMICs). The challenges faced by communities in LMICs, settings with few resources available, can be compared to rural America, where resources are also limited. Common barriers to taking care of colorectal cancer patients in these communities are variation in individual provider behavior, social determinants of health such as poverty, how healthcare systems are set up, and the number of specialty doctors practicing in these communities. This review also highlights how LMICs and rural American communities have addressed these problems, through projects such as training new specialty doctors, collaborating with larger hospitals, and technological innovation. Finally, as an example, we highlight a new colorectal surgery fellowship program that addresses the barriers to providing colorectal cancer care in a low-resource setting at both the individual patient and provider level, all the way to the systems level.

**Abstract:**

**Background/Objectives:** Colorectal cancer remains a significant global health challenge, particularly in resource-limited settings where patient-centered outcomes following surgery are often suboptimal. Although more prevalent in low- and middle-income countries (LMICs), segments of the United States have similarly limited healthcare resources, resulting in stark inequities even within close geographic proximity. **Methods:** This review compares and contrasts colorectal cancer outcomes in LMICs with those in resource-constrained communities in rural America, utilizing an established implementation science framework to identify key determinants of practice for delivering high-quality colorectal cancer care. **Results**: Barriers and innovative, community-based strategies aimed at improving patient-centered outcomes for colorectal cancer patients in low resource settings are identified. We explore innovative approaches and community-based strategies aimed at improving patient-centered outcomes, highlighting the newly developed colorectal surgery fellowship in Sub-Saharan Africa as a model of innovation in this field. **Conclusions:** By exploring these diverse contexts, this paper proposes actionable solutions and strategies to enhance surgical care of colorectal cancer and patient outcomes, ultimately aiming to inform global health practices, inspire collaboration between LMIC and rural communities, and improve care delivery across various resource settings.

## 1. Introduction

Despite well-established guidelines for colorectal cancer (CRC) screening and management, care delivery and outcomes are anything but homogeneous in the United States (USA) and abroad. Colorectal cancer (CRC) is the second leading cause of cancer-related deaths worldwide, with its incidence more than doubling globally over the past three decades. This upward trend is expected to continue, particularly in low- and middle-income countries (LMICs), where the adoption of Westernized lifestyles is contributing to a significant rise in CRC cases [1,2,3]. Similarly, living in rural US areas is associated with a later stage of CRC at diagnosis, lower likelihood of receiving appropriate chemotherapy, and worse survival [4,5,6]. The rural–urban divide in CRC mortality has only widened over time as critical access hospitals have closed [7]. These disparities are not only echoed, but also amplified, when comparing the incidence and mortality of CRC between LMICs and high-income countries (HICs), specifically the United States [8,9,10,11].

Prevention, detection, and treatment of CRC relies on adequate access to reliable healthcare infrastructure, and specifically to specialists trained in modern treatment of the disease. Proposed solutions to improve CRC care often focus on sites where care is already delivered, promoting centralization to high-volume centers; however, such solutions critically fail patients whose primary barrier is access to care at all. In the rural USA, traveling to centralized care may mean securing funds and transportation to cross regional or state lines, leaving social support systems behind; in some LMICs where no specialist exist, this may suggest that patients should cross country or continental borders. Moreover, even if patients were capable of traveling to expertise, centralization without attention to capacity is bound to fail. Communities and health systems in LMICs and low-resourced areas of the USA face similar challenges in advancing CRC care and improving patient outcomes. A deeper understanding of the existing healthcare infrastructure and expertise and their contribution to existing disparities is critical to guiding the development of effective and sustainable solutions. In addition, collaboration is essential for fostering innovation and growth across low-resource settings.

Within this context, this review seeks to illuminate the shared barriers to providing high-quality, patient-centered care in low-resource settings. This analysis applies the Tailored Implementation for Chronic Disease (TICD) checklist, a scientific implementation framework for determining factors that might prevent or enable practice change in healthcare [12]. Further, we aim to showcase successful initiatives that have addressed these obstacles. We specifically highlight a case study of the first CRS fellowship in Sub-Saharan Africa outside of South Africa, which utilized technology, collaboration, and workforce expansion to address many of the barriers identified on the TICD checklist. Finally, we explore opportunities for future growth and collaboration in delivering high-quality care within resource-constrained environments.

## 2. Narrative Review

### 2.1. Barriers to Optimal Colorectal Cancer Outcomes

It is critical to understand the underlying barriers to care in resource-constrained settings. While each community faces unique challenges and needs, there are overarching themes and common barriers to delivering high-quality CRC care in low-resource settings. The TICD framework identifies determinants of practice across seven domains: guideline factors, individual health professional factors, patient factors, professional interactions, incentives and resources, capacity for institutional change, and social, political, and legal factors (Table 1) [12]. These domains encompass factors at both the micro-level—such as individual patient and provider influences—and the macro-level, including governmental and political determinants. Together, they provide a comprehensive review of the factors impacting effective CRC care delivery in low-resource settings.

#### 2.1.1. Guideline Factors

A lack of clear and consistent guidelines, or the data to create them, is a determinant of practice within the TICD framework. In most HICs, regardless of urban or rural locations, there are standardized guidelines for screening and treatment of CRC, with minor variation between national organizations or governing bodies [13,14,15]. Guidelines for CRC screening and care in LMICs are currently less robust, often limited by a lack of quality data and previously low incidences of CRC [16,17]. Limited medical records and cancer databases have been a longstanding constraint in the health systems of LMICs [18,19]. The WHO has identified the lack of surgical data collection and registries as a significant barrier to achieving accessible, safe, and affordable surgical care in LMICs [18]. In CRC specifically, data are similarly limited. Many studies in Sub-Saharan Africa are single-center studies and data gaps in the EMR have been identified as a barrier to improving care [3,20]. On a regional and national level, population-based or pathology-based registries are exceedingly limited, contributing to the dearth of information on the evolving epidemiology of CRC in LMICs to date [1,3].

CRC screening and treatment guidelines are among the determinants of practice with the largest variation between rural settings in the USA and LMIC communities. Although guidelines developed in HICs could theoretically be translated, the reality is that the infrastructure may not exist to implement them. In contrast, US rural areas may be inadequately resourced to deliver guideline-concordant care locally, but healthcare infrastructure exists that should theoretically support implementation of CRC guidelines. Community-specific guidelines for LMICs that account for available technology and specialists are needed, but LMICs often lack the robust data necessary to create community-specific screening guidelines.

#### 2.1.2. Individual Health Professional Factors

The attitudes, behavior, and guideline adoption of individual physicians can have a large impact on CRC delivery. In the US rural setting, rural cancer survivors in Nebraska emphasized local variation in PCP practice as a barrier to gaining information and access to adequate CRC screening [21]. In LMICs, similar trends were seen in qualitative studies; when patients and providers were asked about barriers to CRC screening in Ghana and Mexico, primary care providers (PCPs) were raised as a theme [22,23]. This barrier is likely to be a lack of knowledge, as healthcare providers when surveyed reported a lack of knowledge among PCPs about colorectal cancer screening and national guidelines [22,23]. Further work by Lussiez et. al. focused on physicians alone and identified that in Ghana, where there are now national guidelines for CRC screening utilizing fecal occult blood testing (FOBT), of those interviewed, only a single physician would recommend guideline-concordant screening [24]. This behavior is reflected in observational data, with notably few CRCs in Sub-Saharan Africa being diagnosed via screening endoscopy and few patients reporting ever having discussed CRC screening with their provider [25,26]. While both rural and LMIC communities report individual health professionals’ behavior as a factor, it is much more notable in the qualitative literature from LMIC communities, particularly surrounding implementation of evolving screening guidelines.

#### 2.1.3. Patient Factors

Socioeconomic status (SES), race, and formal educational attainment are individual patient factors that strongly contribute to the CRC care that rural and LMIC patients receive. Rurality has been shown to exacerbate existing racial disparities in CRC outcomes for patients in rural America. For example, Black Mississippians experience both higher CRC incidence (64.9 per 100,000 vs. 50.5 per 100,000) and mortality rates (26.2 per 100,000 vs. 17.5 per 100,000) compared with their counterparts [27,28]. These disparities are further intensified by the challenges associated with rural healthcare access. Rural populations of racial and ethnic minorities, such as non-Hispanic Black, American Indian, and Alaskan Native groups, have also experienced a disproportionate rise in the incidence of early-onset CRC compared with the non-Hispanic White rural population and their urban counterparts [4].

Socioeconomic status can significantly impact rural CRC patients as well, as rural areas have worse socioeconomic deprivation and higher poverty rates (15.4% vs. 11.9%) according to recent census data [29,30]. Increased mortality rates in rural areas have been generally linked to worse SES [29]. Rural cancer survivors reported being more likely to forgo medical care and medication due to cost, in comparison with urban survivors [31]. It can also lead to significant financial stress, given higher rates of self-employment and further travel distances for rural patients [32,33]. Lower SES is associated with decreased likelihood of receiving surgery and chemotherapy in colon cancer and surgery in rectal cancer [34]. Declining SES also correlates with a rising mortality risk for CRC patients [34]. Even in clinical trials with access to standardized treatment, socioeconomic deprivation has been associated with worse survival outcomes in CRC patients [35].

Financial stress is one of the most frequently identified obstacles in LMICs, by both patients and physicians. Surgery can be a financially catastrophic event for individuals living in LMICs, with an estimated 33 million people facing catastrophic health expenditures due to surgery each year [18]. Cost is also a frequently cited reason, both by patients and physicians, for forgoing CRC screening in LMICs. This includes the initial screening as well as the subsequent testing and treatment required if a diagnosis of CRC is made [22,23,24,36]. Surgical patients in LMICs frequently report deciding not to pursue recommended surgical care due to the prohibitive cost, which is often inaccessible and unaffordable, leading to delays in treatment and worsening health outcomes [18,37].

Lower rates of formal educational attainment in LMICs impact the ability of patients to access CRC screening and care. In qualitative studies, healthcare providers have identified a lack of formal education in the patient population as a key barrier to delivering CRC screening in LMICs [22,24]. In one of those cohorts, over 70% of the community participants had obtained only 6 years or less of formal education [22]. In recent prospective studies, higher education levels were significantly associated with higher rates of attending follow-up diagnostic colonoscopies following positive fecal immunochemical screening tests [26]. Educational attainment also significantly impacts outcomes after a cancer diagnosis. In the general cancer population in LMICs, a lack of formal education leaves patients at higher risk of delays in care and barriers to access [6]. In CRC patients specifically, lower education levels have been associated with significantly worse survival [38].

Interestingly, patient factors are also a major determinant of CRC care in rural communities within the United States. While socioeconomic status (SES) is a significant stressor in both LMICs and the rural USA, evidence shows that patients in rural US settings experience worse treatment uptake and survival rates. Both rural American and LMIC patients face challenges in accessing screening and treatment due to socioeconomic factors, as highlighted in qualitative studies. The key divergence between the rural USA and LMICs lies in other patient-level factors. In the USA, the pervasive institutionalized racism within the healthcare system makes race a critical determinant of CRC care. In contrast, race is not a prominent theme in the data from LMICs; instead, formal educational attainment is a more significant factor influencing patient outcomes. Formal educational attainment, which is linked to various health outcomes and is a target of many national and global development goals in LMICs, has not been identified as a significant factor from the rural American perspective. However, for CRC patients and providers in LMICs, it emerges as a strong determinant of practice [39,40,41,42]. Given the high levels of variation in standardized, accessible education across LMICs, there are significant differences in patient-level demographics and their effects on CRC care [39,40,42].

#### 2.1.4. Professional Interactions

Professional interactions—encompassing the professional network, teams, and social influences surrounding a provider—play a crucial role in influencing individual providers’ abilities to deliver high-quality CRC care and their willingness to embrace changes of practice. In HICs, such as the USA, identifiable barriers to delivering colorectal care in rural settings include regional variations in referral and care networks, access to screening, and the reimbursement structure [43]. In a recent mixed-methods study by Crabtree-Ide et. al., stakeholder perspectives on cancer care delivery in rural US communities were collected [43]. Responses highlighted the challenge that regional variations in opinion leaders, local government and programmatic support, and hospital networks have on the implementation of novel programs [43].

A study on Ghanaian physicians’ behavior regarding CRC screening provided valuable insights into how professional interactions act as determinants of practice in LMICs. The study revealed that these physicians had a low uptake of implementing national CRC screening guidelines for their patients [23]. Perhaps even more indicative of the effects of societal influence, none of the physicians aged 50 or older reported undergoing CRC screening themselves. Additionally, a qualitative study on CRC screening identified primary care physicians’ reluctance to change or implement new programs as a significant barrier to improving CRC screening rates [22]. Long wait times for referrals to follow up on positive CRC screening results further highlight the detrimental impact that insufficient professional networks can have on determinants of practice [22]. In both LMICs and the rural USA, the most notable theme in professional interactions is local-level variation. Qualitative data from both settings underscore how this aspect of the TICD checklist is highly susceptible to community factors such as societal values and referral networks.

#### 2.1.5. Incentives and Resources

At the systems level, incentives and resources encompass the physical resources available to providers, including personnel and staff, as well as financial incentives for CRC care. Both LMICs and rural communities face numerous challenges in this area. Providers in LMICs, for instance, often encounter constraints related to equipment and facilities, with not all hospitals equipped to perform FOBT [23]. Physicians additionally note they are unable to follow up positive screening with endoscopy or other testing [23,26]. When the diagnosis of CRC is made after screening, the insurance incentive to treat can be a barrier as well, with national insurance plans lacking coverage for CRC treatment even in countries with national CRC screening guidelines such as Ghana [24].

Physicians and staffing are also critical resources. In LMICs, there is a significant deficit of health workers, which constitutes a major barrier to improving the quality of health services, including CRC care [19,44], and the deficit of specialty care such as that provided by surgeons and anesthesiologists in LMICs is even more. Despite having nearly half the world’s population, LMICs have only 19% of the world’s surgeons and 15% of the anesthesiologists [45]. Not only is there a shortage of general surgeons, but there is also a critical lack of colorectal specialty surgeons, who are essential for the care of CRC patients [46,47,48,49]. This shortage is due in part to the limited availability of training programs; currently, only 9 LMICs offer colorectal surgery fellowship training to develop specialty providers [50]. Due to this paucity of specialists, CRC is often managed by general surgeons in the community [51]. A low density of specialist surgeons means surgical patients in LMICs may be traveling far distances to access care, which could prohibit many from accessing care [52]. In addition, providers practicing in LMICs face a high burden, with work overload frequently cited as an obstacle to delivering higher-quality care [22]. Retention of qualified physicians and other skilled healthcare workers also remains a challenge, due to migration and other economic stressors [44].

A similar determinant of practice is observed in rural America, where higher densities of gastroenterologists, medical oncologists, and surgical oncologists are found in urban areas compared with rural areas—a divide that has widened over the past decade and a half [53,54]. Nearly one in five Americans live in rural areas, yet in 2018, ASCO reported that only 7% of oncologists were based in these locations [55,56]. More than 43 million rural Americans live over 2.5 h away from a primary or satellite National Cancer Institute (NCI) center [57]. This scarcity of specialty providers directly impacts CRC outcomes for rural patients [58,59]. For instance, a geospatial analysis in Pennsylvania revealed that areas with high CRC mortality rates were often located far from ambulatory surgery centers, highlighting disparities in access to care [59].

This demonstrates what is termed a “spatial disparity” between the community’s need for colorectal surgery and screening and the existing infrastructure. Similar studies, including those by Khan et al., show increased CRC mortality in areas with lower densities of colorectal surgeons, even when accounting for social determinants of health [58]. This local shortage of specialty providers forces rural patients to travel long distances to access cancer care [60,61]. Multiple studies report that rural cancer patients travel median distances of around 50 miles for care, nearly eight times greater than their urban or suburban counterparts [60,61]. These longer travel distances have significant impacts on CRC outcomes, including a later stage at diagnosis [6]. Rurality itself is associated with more advanced disease at diagnosis, inadequate lymphadenectomy for stage I–III disease, and decreased likelihood of receipt of adjuvant chemotherapy [5,62].

Reimbursement structures have been identified by rural physicians as a potential determinant of practice. Providers in rural settings are often affiliated with private practices or community hospitals, which are not incentivized to engage in multidisciplinary care to the same extent as their academic counterparts [43,63]. Funding also plays a crucial role in local infrastructure and programming, with rural communities often disadvantaged in competing for mini-grants from state CDC funding due to a lack of pre-existing infrastructure or local non-profit partners [64]. Resource constraints, including the shortage of specialty-trained colorectal surgeons, are some of the most relatable barriers faced by rural communities and those in LMICs. Despite a more robust healthcare and medical education system, rural areas in the USA still experience a deficit in colorectal surgery expertise.

#### 2.1.6. Capacity for Organizational Change

At a higher level of determinants of practice, capacity for organizational change reflects a system’s ability to incorporate new information, its readiness for change, and the responsiveness of leadership. A recent mixed-methods study by Crabtree-Ide et al. highlighted this determinant by illustrating how variations in the opinions of leaders, local government, programmatic support, or hospital networks can impact the implementation of new programs in rural US communities [43].

Similarly, stakeholders in LMICs express concerns about the lack of interest from government or community leaders as a barrier to CRC screening [22,24]. Reports from healthcare personnel and patients in LMICs indicate a pervasive sense of disinterest from local community leaders and national health plans, which hampers CRC care delivery. In both rural and LMIC settings, it is evident that investment from local community leaders, health systems, and sometimes even local governments is crucial for achieving practice change.

#### 2.1.7. Social, Political, and Legal Factors

Finally, legislative constraints, social ideology, and regulations are categorized under social, political, and legal determinants of practice. In LMICs, a lack of prioritization from local governments and inadequate funding for CRC care are significant barriers [22,24]. Patients and providers often report that CRC is not sufficiently prioritized by the government, as evidenced by national health insurance plans that lack coverage for CRC screening and treatment, even in the presence of national CRC screening guidelines [22,24]. Similarly, rural communities face comparable barriers. Regional variations in local government support have been identified as obstacles to implementing new cancer programs [43]. Insurance status is a critical predictor of access to cancer care, with rural Americans being more likely to be uninsured [65,66]. For cancer patients in general, lack of insurance is associated with later stages at diagnosis, and having insurance is linked to lower mortality rates for rural cancer patients [4,6,29].

In both rural US and LMIC communities, political factors and governmental prioritization are related to the delivery of CRC care. Interestingly, despite widely varying economic conditions and other national factors, both rural American patients and those in LMICs struggle with insurance coverage for CRC care. As noted in the patient factors section, the combination of poverty and lack of insurance coverage can turn a CRC diagnosis into a life-altering financial burden.

### 2.2. Areas of Innovation in Delivering High-Quality Colorectal Cancer Care

The question then becomes: What lessons can we learn from these settings, and how can we leverage opportunities to reduce disparities both locally and globally? No single solution will fit all contexts, but by emphasizing exemplary programs we aim to provide a starting point and adaptable strategies for any local context. Three key areas of strategy are highlighted: collaboration and knowledge sharing, technology innovation, and workforce expansion. Additionally, a case study is presented to explore an example of innovation in CRC delivery and demonstrate how these three strategies can address the TICD checklist domains.

#### 2.2.1. Collaboration and Knowledge Sharing

To address individual provider and patient factors, professional interactions, resources and incentives, and capacity for change, various strategies for collaboration and knowledge sharing have been successfully implemented in LMICs and rural communities. A unique aspect of rural healthcare in the USA is the proximity to well-resourced healthcare networks in nearby urban areas. Interviews and insights from leading rural innovators consistently highlight a strong desire for partnerships between local rural healthcare systems and larger accredited or academic cancer centers [67,68,69,70]. Rural cancer providers frequently express a need for these partnerships in order to access resources, educational opportunities, clinical trials, and improved data collection [67,68,70]. Lewis-Thames et al. demonstrated that rural centers can effectively form such partnerships with urban cancer centers, resulting in educational workshops that significantly enhance rural cancer patients’ knowledge about their diagnosis and care [69].

Similar to rural practitioners in the USA, centers in LMICs have also successfully leveraged international partnerships to enhance CRC care quality and delivery. In Rwanda, the Butaro Cancer Center of Excellence has partnered with US academic institutions and NGOs to bolster both financial support and clinical expertise [20,71]. Through these collaborations, the center benefits from structured support provided by US oncologists, significantly strengthening their capacity to deliver high-quality oncological care.

Quality collaboratives also hold promise for improving CRC delivery in rural areas. In states like Michigan, quality collaboratives for oncology and surgical care have led to the creation of working groups focused on colorectal cancer outcomes and increased regional collaboration [72,73]. Similarly, rural health researchers in Mississippi have proposed establishing quality collaboratives in radiation oncology to address rural cancer disparities in the state [74]. The authors of this perspective argue that quality collaboratives could mitigate local variations in treatment, improve accreditation rates through national societies, and enhance partnerships with insurance providers to emphasize value-based reimbursements [74]. Given the strong case for quality collaboration in rural cancer care and successful examples in CRC, further exploration of these collaborations for rural CRC care is warranted.

Patient navigation is a proven tool for reducing disparities in access to care and treatment delays, and its application is being explored in rural CRC care settings [75]. Patient navigation operates on a spectrum, as evidenced by studies from the rural Midwest where lay patient navigators provided phone-based support, including encouragement and logistical assistance with transportation and appointment scheduling, which led to improved completion rates for CRC screening [76]. Similarly, in Hawaii, lay patient navigators delivering unstructured, culturally concordant care significantly increased rates of endoscopic CRC screening through encouragement, reminders, and logistical support [77]. Notably, Georgia has integrated patient navigation into its Community Cancer Screening Programs (CSSPs), targeting colorectal cancer at federally qualified health centers and demonstrating a successful application of this approach in improving CRC care [78]. These professional navigators provided not only encouragement and logistical support but also notifications to patients and providers about upcoming screenings, educational resources, and financial assistance. Via the CSSPs in rural Georgia, professional navigators significantly increased rates of colonoscopy screening and compliance with CRC screening guidelines through their comprehensive support [78].

#### 2.2.2. Technological Innovation

Advances in technology have the potential to enhance CRC care delivery and specifically address determinants of practice such as guideline factors, professional interactions, resources, and incentives. Telemedicine, for example, has emerged as a valuable tool for connecting rural patients to cancer services. Studies like TeleCARE have demonstrated its effectiveness in increasing the uptake of screening colonoscopy, even in the face of other barriers such as financial cost [79]. Remote monitoring of clinical trial patients through telemedicine is another innovative approach that improves access for rural patients [80]. Early data suggest that virtual monitoring is safe and may enhance access to clinical trials for rural patients, a significant step towards equity. Evidence shows that rural and urban patients enrolled in clinical trials have similar outcomes, which may help bridge gaps in cancer care [81].

In response to the dearth of high-quality cancer data in LMICs and the growing use of electronic medical records for simpler data archiving and sharing, there is increasing interest in enhancing cancer data collection in these regions. Recently, the Philippines and Colombia have implemented national and regional hospital-based cancer registries, respectively [82,83]. In the Philippines, this initiative has led to rising engagement from hospitals and improved national data, enhancing their understanding of cancer epidemiology in the country [82]. Similarly, other LMICs such as Rwanda have leveraged international partnerships to develop comprehensive cancer databases [20]. In LMICs with more developed healthcare infrastructures, efforts to link population-based cohorts with cancer registries have expanded the scope of data collection as well [84].

Furthermore, technology and artificial intelligence (AI) hold significant potential for transforming CRC care in LMICs, though further development and local resource investment are required. Telesurgery has been proposed as an innovative method of care delivery in LMICs; however, given that surgery is just one part of the care continuum for CRC patients, the resources required for implementing telesurgery are substantial and may be limiting [85,86]. While telesurgery may face feasibility challenges for direct patient care, its potential for teaching and collaboration is promising and warrants further exploration [85]. Additionally, more accessible technologies, such as AI and machine learning algorithms, offer opportunities to enhance CRC care. Waljee et al. suggest that AI could be utilized in LMICs, including sub-Saharan Africa, to improve the quality and accessibility of CRC care [87]. Technological innovations are of particular interest in low-resource communities, as they often aim to reduce costs and provide access or knowledge not previously accessible to people. Advancements in this field are in the early stages. However, these technologies continue to advance rapidly and further investigation is encouraged.

#### 2.2.3. Workforce Expansion

Finally, focusing on addressing the shortage of skilled healthcare workers and specifically colorectal surgeons will impact determinants of practice such as individual health professional factors, professional interactions, resources and incentives, and capacity for change. This is one of the most promising interventions in low-resource settings, as physicians and skilled healthcare workers represent a sustainable resource with widespread impacts. To expand oncological care provided locally in Vietnam, the National Cancer Control Plan provided training courses to local clinicians on cancer screening and detection [71]. In Rwanda, a local cancer center leveraged international partnerships to enroll Rwandan physicians in oncology fellowships abroad, with contracts to return and practice in Rwanda to expand the local population of oncologists [71]. Local colorectal fellowship training programs are also being developed to enhance the numbers of oncologists in LMICs. The Ghana College of Physicians and Surgeons developed a colorectal surgery fellowship program in collaboration with a US academic center to address the need for colorectal surgical specialists in Sub-Saharan Africa (see Case Study below) [49]. To address issues of retention, the surgical workforce needs early exposure to global rural practice as medical students and loan repayment programs have also been proposed to shore up staffing shortages [18].

Workforce expansion in LMICs also extends beyond physicians to include community health workers (CHWs) and task-sharing strategies. Due to the limited numbers of physicians and skilled healthcare workers, health systems in LMICs have increasingly relied on CHWs to reach patients and communities. In Rwanda, CHWs have been crucial in cancer care delivery, particularly improving the quality of life for cancer patients [88,89]. Additionally, to address the shortage of surgeons, endoscopists, and anesthesiologists, trained medical officers have been explored as a solution for performing endoscopies, with findings indicating that this approach is safe [90]. However, it is important to ensure appropriate training and supervision of these medical officers, as concerns about the ethics of delegating such tasks to non-physicians in LMIC settings have been raised [91].

In rural US settings, the discussion on workforce expansion focuses less broadly on the training of doctors and colorectal surgeons, given the large American healthcare workforce, and instead on attracting and retaining colorectal surgeons in rural settings. There is already a low density of surgeons in rural areas, but with an aging workforce, this need has been forecasted to grow rapidly in the coming years as more rural surgeons retire [92,93]. Medical students have been identified as a key target group for recruitment to rural health posts, with strategies including increased mentorship from rural surgeons and enhanced exposure to rural surgical careers proposed to boost interest in rural practice [94]. General surgery residents represent an even more targeted population for intervention. Proposals include creating rural surgery-specific tracks with broad caseloads and larger case volumes to better prepare residents for the demands of rural surgical practice [94,95,96,97]. Attention and funding have also been directed toward increasing residency positions in rural hospitals [92,98].

## 3. Case Study

We present the Ghana College of Physicians and Surgeons Colorectal Surgery Fellowship Program as an exemplar intervention in addressing barriers to high-quality CRC care delivery in an LMIC. In Ghana, the absence of specialized colorectal surgery (CRS) providers has been a significant barrier to timely diagnosis and effective healthcare delivery. For instance, the five-year survival rate for patients with colorectal cancer (CRC) in Ghana is only 16%, compared with 65% in the United States, for all stages combined [99,100].

In response to the urgent need for specialized colorectal care in Ghana, the Ghana College of Physicians and Surgeons (GCPS), in collaboration with key teaching hospitals across the country and supported by colorectal surgeons from the United States, launched Ghana’s first Colorectal Surgery Fellowship Program in 2023. This multi-institutional initiative draws on resources and expertise from various academic and clinical centers within Ghana, with mentorship and curriculum support provided by international colorectal surgeons and professional societies.

The fellowship is designed to equip surgeons with advanced skills to manage complex colorectal diseases in Ghana, emphasizing both clinical excellence and academic leadership. Fellows receive comprehensive training in minimally invasive techniques, multidisciplinary care coordination, and patient-centered approaches to colorectal disease management. As part of their training, fellows undertake a one-month externship at a high-volume colorectal surgery tertiary center in the USA. During this externship, they gain experience in advanced surgical techniques and learn how to establish and manage comprehensive care pathways, often involving a multidisciplinary approach. Their externship culminates in attending the annual national colorectal society meeting in the USA, where they network with peers and experts from both the USA and other global regions.

To support continued medical education, virtual meeting platforms provide bi-monthly training sessions for CRS fellows and interested Ghanaian general surgeons. Additionally, the program in Ghana has explored using hands-free, voice-activated, HIPAA-compliant headgear to facilitate teaching bedside and operative content to learners in more remote locations. Upon successful completion of the fellowship program, fellows are recruited to join the teaching faculty, ensuring a sustainable pipeline of skilled colorectal surgeons capable of addressing the growing burden of colorectal diseases in Ghana and the broader West African region.

**Figure 1:**
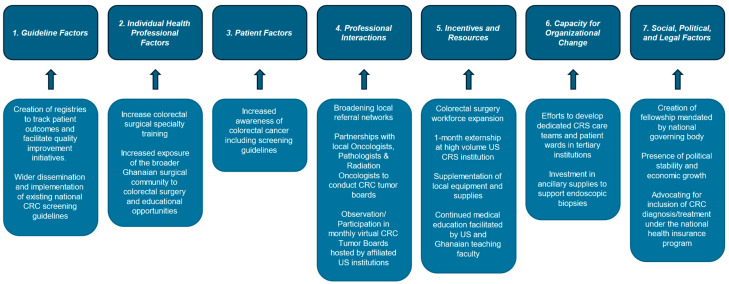
Ghana College of Physicians and Surgeons Colorectal Surgery Fellowship Program mechanisms of action within the 7 domains of Tailored Implementation for Chronic Diseases.

These efforts target all seven domains of the TICD checklist, as illustrated in Figure 1, utilizing collaborative knowledge sharing, technological advances, and workforce expansion. The program has not only improved access to timely and specialized colorectal care but has also established a robust framework for the continued development of colorectal surgery as a discipline within the country. Since the fellowship’s establishment, more patients have received life-saving interventions that were previously inaccessible, improving survival rates and overall patient outcomes. Additionally, efforts are underway to create registries to track patient outcomes and facilitate quality improvement initiatives.

## 4. Future Directions

Further progress in this area depends on continued collaboration between patients, community stakeholders, healthcare systems, and their leaders. This paper highlights the similar challenges faced by rural American and LMIC communities and suggests that future research should focus on bridging these disparate yet comparable groups. Notable innovations emerging from these settings include the use of technology such as artificial intelligence for improved diagnostics and telesurgery for overcoming geographical barriers in care delivery. Additionally, enduring strategies that will remain crucial for both rural US and LMIC settings involve community-driven partnerships, investment in and development of the skilled healthcare workforce, and the expansion of insurance coverage. Addressing these barriers with innovative and community-based interventions is increasingly important as the global landscape of colorectal cancer continues to evolve.

## 5. Conclusions

By exploring these diverse contexts, this paper proposes several solutions and strategies to enhance CRC surgical care and patient outcomes, ultimately aiming to inform global health practices and improve care delivery across various resource settings. Common barriers to delivering high-quality CRC care in both the rural USA and LMICs include limited access to local specialty surgical care and the influence of socioeconomic disparities on patient outcomes. In both settings, patients often reside far from cancer centers, which affects screening, access to care, and overall CRC outcomes [51,53,54,59]. Interestingly, both settings have also leveraged partnerships with more established cancer centers, whether urban accredited cancer centers in the rural US setting or international accredited cancer centers in the LMIC setting, to increase access to specialists and patient education [69,71]. This theme was frequently highlighted by rural oncology providers in the USA as a desired improvement for their patients’ cancer care. Overall, such partnerships offer tangible benefits, including workforce training and the implementation of cancer databases in LMIC settings [67,68].

Socioeconomic disparities, particularly poverty, have played significant roles in hindering access to care and negatively impacting outcomes in both settings. As a population-level variable, this is challenging to modify effectively through interventions, and no common strategies have been identified for addressing this barrier. However, a related factor emerges in both settings: insurance status. Qualitative studies on barriers to care have frequently highlighted insurance status and coverage of CRC services for both rural patients and those in LMICs [6,36,62,66]. Expanding Medicaid/Medicare in the USA and increasing insurance coverage in LMICs are potential avenues for improving access to CRC services.

## Figures and Tables

**Table 1:** Barriers to delivery of high-quality colorectal cancer care in LMICs and rural communities in the USA, as categorized by the 7 domains of the TICD checklist with notable areas of alignment and divergence.

	LMICs	Rural US Communities	Alignment	Divergence
**1. Guideline Factors**	Limited cancer databases; few national guidelines for CRC screening and care.			Wide differences in quality of cancer databases and availability of national or applicable global CRC guidelines.
**2. Individual Health Professional Factors**	Lack of PCP knowledge of CRC screening guidelines.	Local variation in PCP practice	Variation in PCP knowledge and implementation of CRC screening guidelines.	More frequently noted in LMICs than the rural USA in the literature.
**3. Patient Factors**	Low SES status; formal educational attainment.	Low SES status; racial minority.	Low SES status.	Impact of institutionalized racism in the USA.
**4. Professional Interactions**	Low implementation of CRC guidelines among physicians; insufficient professional /referral networks.	Regional variation in social and administrative support for change; regional variations in referral networks.	High reported levels of regional variation; impact of referral networks.	Low implementation of guidelines in LMICs compared with more noted impact of varying support in rural communities.
**5. Incentives and Resources**	Colorectal surgeons as a scarce resource equipment and physical resource shortages; endoscopy shortage.	Colorectal surgeons as a scarce resource; impact of reimbursement structure on physician behavior.	Staffing shortages and low density of colorectal surgeons.	Impact of the constraints of broader health system shortages in LMICs, with endoscopy limitations and supply shortages.
**6. Capacity for Organizational Change**	Lack of interest from government or community leaders.	Lack of interest from government or community leaders.		Investment from local community leaders, health systems, and sometimes even local governments is crucial for achieving practice change.
**7. Social, Political, and Legal Factors**	Lack of coverage for CRC under national health insurance plans.	Negative impact of being uninsured.	Insurance status and coverage is impactful.	
